# A Comparative Analysis of Optimal Cutting Temperature (OCT) Compound and Water as Embedding Media in Frozen Section

**DOI:** 10.5146/tjpath.2026.14824

**Published:** 2026-05-30

**Authors:** Umesh Sharma, Puneet Kaur Somal, Aishwarya Sharma, Nilay Nishith, Rahul Raj, Ravikiran Pawar, Sankalp Sancheti

**Affiliations:** Department of Oncopathology, Homi Bhabha Cancer Hospital and Research Centre, Homi Bhabha National Institute (HBNI), PUNJAB, INDIA; Department of Oncopathology, Homi Bhabha Cancer Hospital and Research Centre, Homi Bhabha National Institute (HBNI), Punjab, India

**Keywords:** OCT compound, Frozen section, Water, Embedding media

## Abstract

Objective: The commonly used embedding medium in frozen section is a commercially available optimal cutting temperature (OCT) compound, which is a water-soluble mixture of glycols and resins. However, its high cost limits its widespread use in laboratories with limited resources. In the present study, we compared two embedding media (OCT compound & Water) based on the mean freezing time and effect on the quality of section in terms of staining and the presence of freezing artifacts in different types of tissues.

Material and Methods: Fresh & unfixed specimens from 45 cases were analysed prospectively. The specimens included mucosal margin assessment (n=15), lymph node metastases (n=16), and fatty tissues (breast lumpectomy) for margin assessment (n=14). Average freezing time, presence of freezing artifact (extensive, minimal, or absent) and quality of nuclear and cytoplasmic staining (good, satisfactory, or poor) were noted.

Results: Overall mean freezing time was marginally lower for OCT (3’34”) when compared to water. (3’58”) Lymph nodes took the shortest time to freeze irrespective of the embedding medium used. Fatty tissues took the longest time to freeze with water (4’12”) as compared to OCT (3’35”). With OCT, the mean freezing time was lowest for lymph nodes (3’22”) and highest for mucosal margins. (3’45”) Extensive freezing artifacts were more commonly observed with OCT than water. (37.8% vs 31.1% respectively) Staining quality was comparable for both embedding media overall and amongst the different types of tissues.

Conclusion: In settings where cost is a limiting factor, water can function as a cheaper and readily available embedding alternative to OCT. In frozen sections involving fatty tissues where time is a limiting factor, OCT provides a shorter mean freezing time and can be used in place of water.

Keywords: OCT compound, Frozen section, Water, Embedding media

## Introduction

Frozen section technique is one of the most definitive forms of intraoperative consultation and plays an important role in optimizing the surgical management of cancer patients. Its utility is manifold ranging from identification of a pathological process, adequacy of margins of surgery, and identification of lymph node metastases with studies reporting an accuracy ranging from 85 to 90% (1).

The tissue is frozen rapidly and put onto the cryostat for sectioning. Intracellular water is frozen to produce a hard matrix that enables slicing of the tissue (1,2). Water-containing tissue is usually sectioned at higher temperatures and fat-containing tissue is sectioned at lower temperatures (3). Rapid freezing of the tissue sample converts the water into ice and acts as a medium that aids the subsequent sectioning of the tissue. Lowering the temperature makes the tissue firmer, whereas increasing temperature makes the tissue softer (3).

The commonly used embedding medium is a commercially available optimal cutting temperature (OCT) compound, which is a water-soluble mixture of glycols and resins (3). It facilitates easy cutting of frozen sections in the cryostat. It leaves no residue on slides during staining, which eliminates undesirable background staining. Fresh-frozen, bone marrow aspirates embedded with OCT compound have been found to be a reliable resource for morphological, immunohistochemical and molecular examinations (4). A major limitation in its use in resource-poor settings is the relatively high cost that hampers sustained supply. Thus, there is a need for an alternative.

In a study by Sahabi et al., the authors sought to identify a cheaper alternative to OCT compound. They compared commercially available OCT compound with readily available and easy to handle alternatives such as water and office glue. They found comparable results with OCT compound and office glue. Freeze and staining artifacts were minimal in tissues processed using OCT compound and glue in comparison to water (2).

Hye Kyung Baek et al. tested a set of conditions for obtaining optimal tissue quality in preparation for histology in samples of mouse brains. Half of the brain tissues were embedded in OCT compound and half were not embedded. The authors observed more freezing artifacts in OCT compound embedded brains in comparison to the non embedded tissues (5). We sought to compare water and optimal cutting temperature (OCT) compound as embedding media based on the effect on the quality of staining and the presence of freezing artifacts.

## Materials and Methods

The present study was conducted over a one-year period from April 01, 2022, to March 31, 2023. Adequately labelled fresh & unfixed specimens received for intra-operative consultation for common oncological indications like margin assessment in fatty tissues like breast lumpectomy specimens, mucosal margin assessment in non-fatty tissues like head and neck surgical specimens (e.g., hemi mandibulectomy, tongue/lip/buccal mucosa wide excision), and lymph node assessment for metastases were included in the study. Biopsy specimens and calcified tissues were excluded.

**Figure 1:**
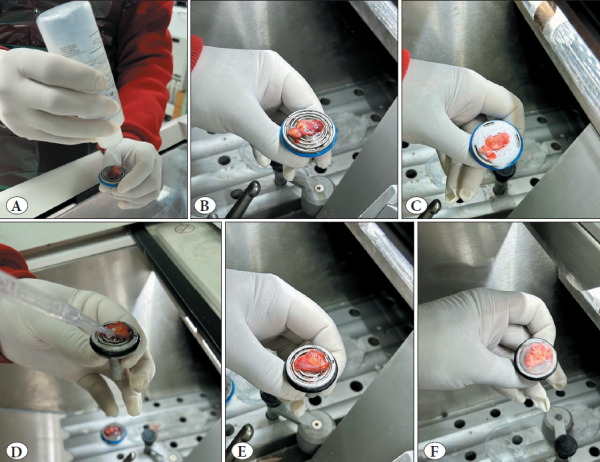
Tissue section taken on a chuck with an adequate amount of OCT Compound (A,B) & water (D,E). The chucks were then transferred to the cryostat till the tissue was completely frozen (C using OCT compound & F using water).

The unfixed frozen tissue sample was received in the laboratory from the operation theatre with a request form signed by the operating surgeon. The frozen tissue sample was subsequently grossed by a pathologist and two representative sections were taken. One tissue section was taken on a chuck and an adequate amount of OCT compound was poured to ensure adherence of the tissue to the chuck Figure 1. The OCT compound used was Epredia cryomatrix, Fisher Scientific (USA) with the following composition-Water (80-85%), Polyvinyl alcohol (10-15%), Polyethylene glycol (2-5%), Potassium formate (1-3%).

The same procedure was repeated on another chuck with water. Figure 1 The chucks were then transferred to the cryostat and freezing time was calculated for each medium. Freezing time was defined as the time calculated from placing the chuck into the cryostat till the tissue in the chuck was completely frozen. Once the tissue was completely frozen, sections were cut in the cryostat at 6-8 micron by the laboratory technician and placed on a slide. The slide was stained by the rapid Hematoxylin & Eosin (H & E) staining method.

The stained slides were examined by the pathologist and the parameters studied included the presence of freezing artifact (assessed as extensive, minimal, or absent), and quality of nuclear and cytoplasmic staining (assessed as good, satisfactory, or poor). Descriptive statistics were performed to calculate the average freezing time in each method and to assess the staining aspect of each method.

## Results

A total of 45 cases were included in the present study. The tissues were divided into fatty tissue (margins in breast lumpectomy, n=14), non-fatty tissue (mucosal margin in head and neck surgical specimens, n=15), and lymph nodes (n=16).

The mean freezing time was marginally lower for OCT (3’34”) when compared to water (3’ 58”). It was observed that fatty tissue took the longest time to freeze when using water as the embedding medium (4’12”) while lymph nodes took the shortest time (3’ 24”).

Using OCT as the embedding medium, the mean freezing time was lowest for lymph nodes (3’ 22”) and highest for mucosal margins (3’ 45”). These findings are summarized in Table 1.

**Table 1:** Comparison of mean freezing time (min) for all the tissues.

**Type of tissue**	**Embedding medium (OCT)**	**Embedding medium (Water)**
Fatty tissue (Margin in breast lumpectomy)	3’ 35”	4’12”
Non fatty tissue (Mucosal margin in head and neck surgical specimens)	3’ 45”	3’ 36”
Lymph nodes	3’22”	3’ 24”

### Freezing Artifacts

**Figure 2:**
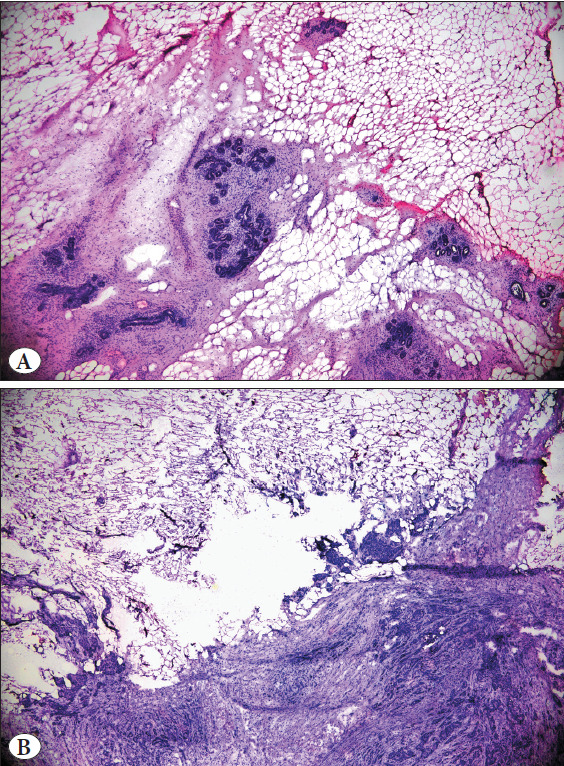
1: Fatty tissue: Minimal artifact in fatty tissue with OCT, 100x (A), Formation of multiple holes in fatty tissue with water,40x (B).

**Figure 3:**
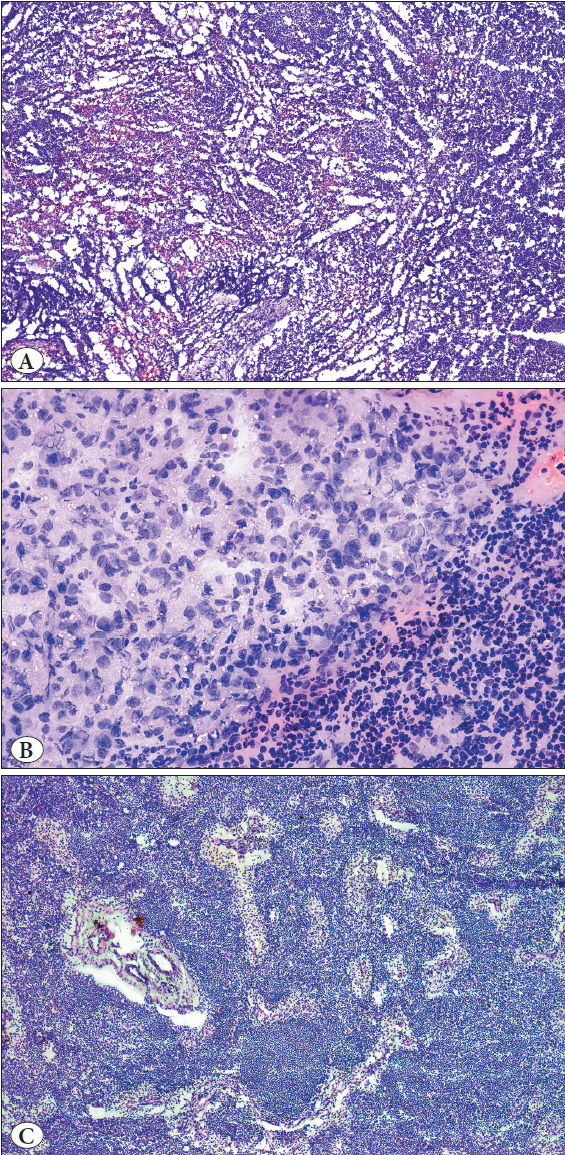
1: Lymph nodes: Ice crystal artifact in lymph node with OCT, 100x (A), Nuclear drying artifact in lymph node with OCT, 400x (B), Minimal freezing artifact using water, 100x (C).

**Figure 4:**
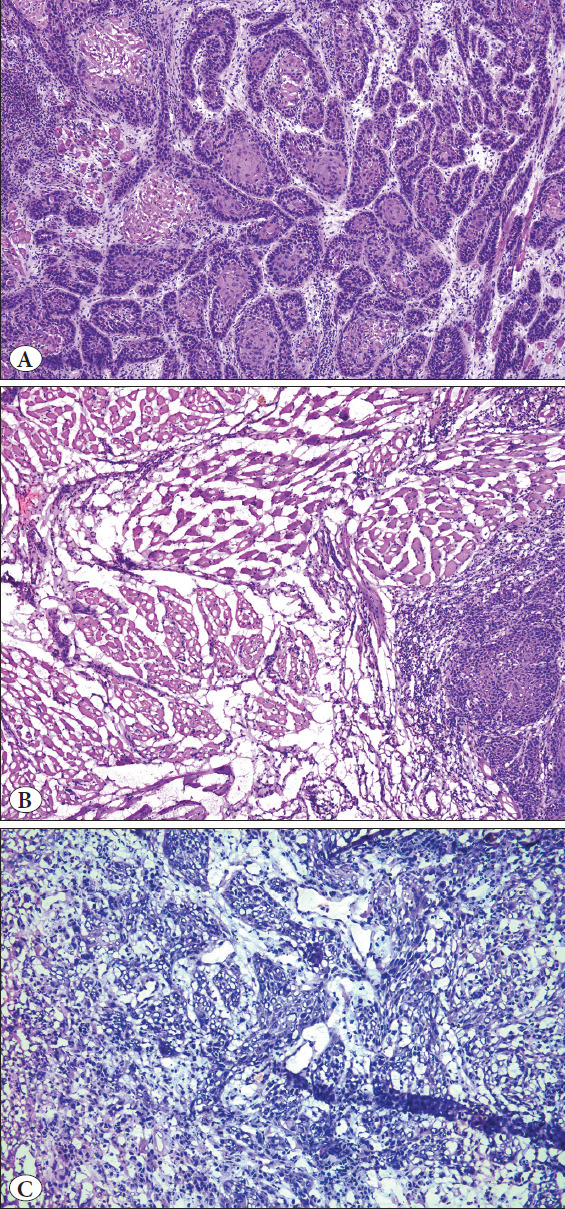
1: Mucosal margins (non-fatty tissue): Good nuclear and cytoplasmic staining in mucosal margin with OCT, 200x (A), Ice crystal artifact in mucosal margin with water, 200x (B), Nuclear holes in mucosal margin using water, 400x (C).

Extensive freezing artifacts were more commonly observed while using OCT as embedding medium in comparison to water (37.8% vs 31.1% respectively). Minimal freezing artifacts were observed in 26 cases with OCT (57.8%) Figure 2 and 28 cases with water (62.2%) Figure 3. Common freezing artifacts included ice crystal artifacts (Figure 2, Figure 3,Figure 4), compression artifacts, drying artifacts (leading to enlargement of nuclei with fuzzy chromatin staining, cytoplasmic vacuolation), nuclear holes (Figure 3, Figure 4), tissue folds, and air bubble entrapment.

The quality of nuclear staining was comparable for both methods with good nuclear staining observed in 20 cases (44.4%) with OCT Figure 4 and 22 cases (48.9%) with water. Satisfactory nuclear staining was seen in an equal number of cases (19) for both methods. Poor nuclear staining was seen in slightly more cases of OCT in comparison to water (13.3% vs 8.9% respectively).

The quality of cytoplasmic staining was also comparable for both methods. Good cytoplasmic staining was observed in 21 cases (46.7%) with OCT and 20 cases (44.4%) with water. Poor cytoplasmic staining was seen in slightly more cases of OCT in comparison to water (20% vs. 13.3% respectively).

Further, there were no discrepancies between the frozen section diagnosis and the final diagnosis in all the cases for both the techniques. The comparison of all the parameters amongst the different tissue types (fatty tissue, non-fatty tissue and lymph nodes) is summarized in Table 2.

**Table 2:** The comparison of all the parameters amongst the different tissue types

-	**Fatty Tissue**		**Non Fatty Tissue**		**Lymph Nodes**	
** Embedding media** **Parameters**	**OCT** **(n=14)**	**Water (n=14)**	**OCT** **(n=15)**	**Water** **(n=15)**	**OCT (n=16)**	**Water** **(n=16)**
**Freezing artifacts**	-	-	-	-	-	-
Extensive	6/14 (43.0)	6/14 (43.0)	3/15 (20.0)	3/15 (20.0)	8/16 (50.0)	5/16 (31.0)
Minimal	7/14 (50.0)	8/14 (57.0)	11/15 (73.0)	9/15 (60.0)	8/16 (50.0)	11/16 (69.0)
Absent	1/14 (7.0)	0/14	1/15 (7.0)	3/15 (20.0)	0/16	0/16
**Quality of nuclear staining**						
Good	5/14 (36.0)	6/14 (43.0)	8/15 (53.0)	10/15 (67.7)	7/16 (44.0)	6/16 (37.5)
Satisfactory	6/14 (43.0)	6/14 (43.0)	7/15 (47.0)	5/15 (33.3)	6/16 (37.5)	8/16 (50.0)
Poor	3/14 (21.0)	2/14 (14.0)	0/15	0/15	3/16 (19.0)	2/16 (12.5)
**Quality of cytoplasmic staining**						
Good	5/14 (36.0)	5/14 (36.0)	9/15 (60.0)	10/15 (67.7)	7/16 (44.0)	5/16 (31.0)
Satisfactory	5/14 (36.0)	7/14 (50.0)	5/15 (33.0)	5/15 (33.3)	5/16 (31.0)	7/16 (44.0)
Poor	4/14 (28.0)	2/14 (14.0)	1/15 (7.0)	0/15	4/16 (25.0)	4/16 (25.0)

Values are presented as n (% within each subgroup).

## Discussion

The present study was undertaken to compare water and optimal cutting temperature (OCT) compound as embedding media in the frozen section. Fatty tissues took the longest time to freeze when using water as the embedding medium.

It is well known that fatty tissues pose a challenge while cutting frozen sections as fat does not freeze easily and does not adhere well to the embedding medium. This can be ameliorated by taking a thicker section (6–8). Using OCT as an embedding medium, fatty tissues had a shorter freezing time with a comparable distribution of freezing artifacts and nuclear and cytoplasmic staining to water. Thus, OCT can be used in cases where time is an issue during the frozen section.

Freeze artifact refers to morphological variations in the microanatomy that result from ice crystal formation as water freezes in the tissue (3). Tissues with high water content, such as the brain and edematous tissues, often yield ice crystals during the actual freezing in the cryostat and result in artifacts (7). Artifacts may occur during tissue processing, embedding, microtomy, and staining and mounting procedures. The common types of artifacts include ice crystal formation, folds, incomplete staining, air bubbles, nuclear drying artifacts (enlargement of nuclei, pallor of chromatin and non uniform eosinophilia of the cytoplasm). Artifactually crushed cells can hamper accurate morphological assessment of tumor (7).

Extensive freezing artifacts were slightly more common with OCT in comparison to water. (37.8% vs 31.1%) In another study done by Miller et al., to assess the preparation of frozen sections for MOHS micrographic surgery, the authors also observed more ice crystal artifacts with OCT (9). Baek et al. also reported more freezing artifacts in OCT compound-embedded tissues in their study (5).

This finding however, contrasts with the observations made by Sahabi et al. They compared commercially available OCT with alternatives such as water and office glue. Freezing artifacts were minimal in tissues processed using OCT and glue in comparison to water (2).

Polyethylene glycol (PEG) is a common constituent of OCT and is widely used as a plasticizer. It has proved to be effective in preventing fractures and cracks between tissue components thereby resulting in reproducible sectioning (8). This has also been reiterated in the study by Reserva et al (10). The authors compared the quality of frozen sections prepared using three commercially available embedding media and observed more efficient sectioning in the media with a higher PEG concentration. The reason for more extensive freezing artifacts with OCT in the present study could be attributed to variability in the cutting of the different tissues. The study done by Sahabi et al. included very few cases of fatty tissue.

Amongst the different types of tissues, we observed that the softer, non-fatty tissue like mucosal margins had the least proportion of cases exhibiting extensive freezing artifacts irrespective of the embedding medium used. On the contrary, the proportion of cases with extensive freezing artifacts was higher for both fatty tissues and lymph nodes. The higher proportion of extensive freezing artifacts in lymph nodes could be due to inadequate trimming of fat around the lymph node with the resultant fat preventing proper adhesion of the tissue to the embedding medium and issues in cutting (5).

OCT is the commonly used embedding medium in frozen sections; however, in a resource-limited setting, the relatively high cost (around 1200-1400 TRY per bottle for 60-70 uses) can prove to be a hindrance. In such a scenario, water can function as a cheaper, readily available, and reliable alternative. One of the disadvantages with water was that the frozen tissues were comparatively harder to cut in comparison to OCT compound with fatty tissue being the hardest. This can be attributed to the increased propensity of water to form either hexagonal or cubic crystals as it freezes and turns solid (2).

One of the main limitations of the current study is the smaller number of cases included under each type of tissue. This precludes observation of more significant differences (if any) amongst both the embedding media. Larger scale studies are thus required for further validation.

## Conclusion

Preparation of frozen section slides is a complex process requiring technical expertise as an improperly cut section can impact the final diagnosis. While OCT is the commonly used embedding medium in frozen sections, in settings where cost is a limiting factor water can function as a cheaper and readily available alternative. In frozen sections involving fatty tissues where time is a limiting factor, OCT provides a shorter mean freezing time and can be used in place of water.

## Conflict of Interest

The authors have no conflict of interest.

## Funding

No financial and material support was received.
